# Excess of Organic Carbon in Mountain Spruce Forest Soils after Bark Beetle Outbreak Altered Microbial N Transformations and Mitigated N-Saturation

**DOI:** 10.1371/journal.pone.0134165

**Published:** 2015-07-31

**Authors:** Jiří Kaňa, Karolina Tahovská, Jiří Kopáček, Hana Šantrůčková

**Affiliations:** 1 Institute of Hydrobiology, Biology Centre of the CAS, v.v.i., České Budějovice, Czech Republic; 2 Department of Ecosystem Biology, Faculty of Science, University of South Bohemia in České Budějovice, České Budějovice, Czech Republic; Chinese Academy of Sciences, CHINA

## Abstract

Mountain forests in National park Bohemian Forest (Czech Republic) were affected by bark beetle attack and windthrows in 2004–2008, followed by an extensive tree dieback. We evaluated changes in the biochemistry of the uppermost soil horizons with the emphasis on carbon (C) and nitrogen (N) cycling in a near-natural spruce (*Picea abies)* mountain forest after the forest dieback, and compared it with an undisturbed control plot of similar age, climate, elevation, deposition, N-saturation level, and land use history. We hypothesised that the high litter input after forest dieback at the disturbed plot and its consequent decomposition might influence the availability of C for microorganisms, and consequently, N transformations in the soil. The concentrations of dissolved organic C (DOC) and N (DON) in soil water extracts rapidly increased at the disturbed plot for 3 yeas and then continually decreased. Net ammonification exhibited a similar trend as DOC and DON, indicating elevated mineralization. Despite the high ammonium concentrations found after the forest dieback (an increase from 0.5 mmol kg^-1^ to 2–3 mmol kg^-1^), net nitrification was stable and low during these 3 years. After the DOC depletion and decrease in microbial biomass 5 years after the forest dieback, net nitrification started to rise, and nitrate concentrations increased from 0.2–1 mmol kg^-1^ to 2–3 mmol kg^-1^. Our results emphasize the key role of the availability of organic C in microbial N transformations, which probably promoted microbial heterotrophic activity at the expense of slow-growing nitrifiers.

## Introduction

Bark beetle outbreaks are important disturbance factors for forest ecosystems, and are common in Europe [1,2], and North America [3–5]. The effects of such disturbances on forest soils include changes in the chemistry of upper soil horizons [6], as well as changes in soil microbial activity [7,8]. The most intensive microbial transformations occur in the uppermost organic-rich soil horizons [9,10]. Their chemistry usually reflects changes in the composition of atmospheric deposition [11], vegetation type [12], and ecosystem disturbances, associated with tree harvesting or natural forest dieback [6,13,14]. Important soil parameters sensitive to ecosystem disturbances include the availability of inorganic N forms and rate of N mineralization processes [7,15–17].

N mineralization is inevitably linked to soil microbial biomass and its ability to immobilize N. Therefore, effects of forest disturbances on soil microbial biomass are to be expected. However, information concerning the coupled response of microbial biomass and N transformations after forest dieback is scarce. Ferrenberg et al. [18] found no significant changes in microbial biomass within 5 years following the bark beetle-induced tree mortality in an area that was not N-saturated, and suggest resistance of soil microbial communities to disturbance of the forest canopy. In contrast, a significant decrease in ectomycorrhizal fungal biomass, as well as a decline in fungi-to-bacteria ratio occurred in soils of the N-saturated Bohemian Forest catchment after the bark beetle-induced forest dieback [8].

In general, forest disturbances enhance the concentrations of mineral N in soil solutions and nitrate (NO_3_) leaching [15, 19–21]. This response is not, however, identical across localities. While a rapid increase in soil ammonium (NH_4_) concentrations has been denoted a universal response to forest dieback [4], enhanced NO_3_ leaching may be negligible or delayed for several years [7,16]. Bengtsson et al. [22] suggested that the appearance of NO_3_ leaching from forest soils may be largely dependent on a decrease in the activity of the soil microbial community.

Trees infested by bark beetles quickly die, and within a one- to two-year period lose non-senescent needles, which are richer in nitrogen, phosphorus and available organic compounds compared to common litter from healthy trees [23,24]. This massive short-term litterfall increases the carbon and nutrient supply to soil microbial communities. In general, microbial activity is enhanced and organic matter decomposition accelerates after the input of fresh organic material to forest floor [25]. In the first stages of decomposition, available N exceeds microbial N demand and N remains in organic soil horizons as organic N or NH_4_ [26]. At the same time, the elevated resources of available C are used in assimilative and dissimilative microbial processes. The active community of heterotrophs occupies most of this niche and can limit the development of slowly growing nitrifiers, thus delaying NO_3_ production, and leaving NH_4_ in the soil [27]. Later, when fresh litter inputs are diminished, easily available C is depleted, relative C and N availability is shifted towards excess N, and consequently the N-limited microbial processes can be switched to C-limited processes [28]. Under such conditions, the activity of heterotrophs decelerates, their competitiveness decreases, and the activity of nitrifying bacteria is promoted, due to abundant NH_4_ available in the system. Even though nitrate is produced in the soil during this phase, it cannot be effectively used in reductive processes due to the energy and C-limitations of the microbial biomass [29], and is leached from the soils. The transition of a system to C-limitation and subsequent nitrate leaching thus results from decreased C availability and C:N stoichiometry of the available substrate for the microbial community, and its decreased ability to immobilize N [30]. Thus, we hypothesise that changes in C availability after forest dieback can alter key mechanistic processes such as N transformations and therefore affect the rate of NO_3_ leaching from near-surface forest soils.

To test this hypothesis, we performed an intensive six-year (2008–2013) sampling campaign at sites either affected or unaffected by a bark beetle infestation. The investigated spruce forest ecosystems in the Bohemian Forest (Czech Republic) are N-saturated due to high and long-lasting atmospheric depositions of inorganic N during the 20^th^ century [31]. We hypothesized that soils in forests where all the trees were killed by bark beetles could pass through the short period of C surplus due to the transient increase in C availability after the elevated litterfall, and then return back to a relatively C-limited (and N-saturated) status. We measured changes in the microbial biomass (microbial C and N), net nitrification and ammonification, and concentrations of DOC and N forms in soils to answer the following questions: (1) What are the effects of a natural forest dieback on soil C and N availability in N-saturated mountain spruce forest soils? (2) What is the role of organic C in alteration of microbial N transformations?

## Material and Methods

### Study area and sampling strategy

Both study plots are situated in the Bohemian Forest National Park, a central European mountain area, with unmanaged forests and limited human activities since the Second World War. One exception was a high atmospheric deposition of S and N compounds up to 1980´s, which caused acidification and N-saturation of the study area [31,32]. The study was performed at two plots (50×50 m) situated in the catchments of Plešné (PL; N 48.7752, E 13.8680) and Čertovo (CT; N 49.1627, E 13.1993) lakes (distance between the plots is 65 km). Both plots are situated in the lower parts of the PL and CT catchments at altitudes of 1122 and 1057 m a. s. l., respectively; long-term average temperatures and annual precipitations are similar at both plots. The unmanaged forests in the PL and CT catchments are on average ~160 and 90–150 years old, respectively, and are dominated by Norway spruce. For more details on the study plots, forest and land use history see Ref. [33–38]. The forest at the PL catchment has been completely killed by a bark beetle infestation, affecting the area from 2004–2008 and starting at the PL plot in 2006 [7]. The forest at the CT plot was damaged only negligibly by a windstorm in 2007 [33].

The PL and CT bedrocks are composed of granite and mica-schist, respectively. Soils at both plots are spodo-dystric cambisols and podsols [36].

More details on the chemistry of upper soil horizons (pH, base saturation, exchangeable cations and acidity) at the study plots are given by Kaňa et al. [6]. That study also evaluated detailed changes in soil chemistry during the first three years (2008–2010) following the bark beetle infestation, especially in the concentrations of NH_4_, DOC, PO_4_-P, base cations, and ionic Al during the early stages of forest dieback.

Soils were sampled under the tree canopy in six-week intervals (8–9 samplings per year) from January 2008 to December 2013 (also under snow cover, usually present from November/December to March/April). For the purpose of this study, we use the following classification of soil horizons: the forest floor horizon (O; Ol + Of) and the uppermost organic-rich mineral horizon (A). Samples were taken separately from each soil horizon in six pits (15×15 cm, 225 cm^2^) and were weighed to determine soil pools (kg m^-2^) in the horizons. After each sampling, we prepared mixed samples for each soil horizon by combining samples from 2 randomly selected pits. Thus, analyses were performed for 3 mixed samples of both O and A horizons for each sampling, and the results further presented are averages for these three mixed samples.

Samples were stored at 4°C in the dark until analysed (3–5 days). Prior to the analyses, soil samples were passed through a 5-mm stainless-steel sieve to remove coarse particles and divided into two parts. One part (with moisture level as taken in the field) was analyzed for microbial biomass, N transformation, and concentrations of water-extractable dissolved organic carbon (DOC) and N forms. The other part of the soil sample was air dried between two sheets of filter paper for 14–21 days at laboratory temperature, sieved through a stainless-steel 2-mm sieve, and used for the remaining chemical analyses. This air-dried <2-mm soil fraction is further referred to as the AD soil.

### Chemical analyses

Subsamples of the AD soil for the purpose of elemental analyses were finely ground to pass through a 100 μm sieve. The concentrations of total C and N were analyzed using a CN analyzer (ThermoQuest, Italy). The concentrations of total P were measured colorimetrically after nitric and perchloric acid digestion [39]. Water extracts (1:10 by weight; field moist soil; 1 hour shaking on a horizontal shaker; filtration through glass fibre filters, Macherey-Nagel, 0.4 μm porosity) were analyzed for water-extractable DOC, total dissolved nitrogen (TN_H2O_), NO_3_, and NH_4_. The concentrations of NH_4_ and NO_3_ were measured colorimetrically using a flow injection analyzer (FIA) consisting of a FIA Star 5027 Sampler, 5012 Analyzer, and 5042 Detector (Foss Tecator, Hoganas, Sweden) [40] after filtration of the samples through Whatman GF/C filters. The gas diffusion method was used to determine NH_4_ [41]. NO_3_ was determined after reduction to nitrite. The concentrations of DOC and TN_H2O_ were analyzed with a Formacs TOC/TN analyzer (Skalar, Netherlands). The concentration of dissolved organic nitrogen (DON) was measured as the difference between TN_H2O_ and the sum of NO_3_ and NH_4_. The pH was measured in a 1M KCl solution (pH_KCl_), with a mass ratio of the AD soil to liquid phase of 1:10 after a 2.5-hour extraction (horizontal shaker). The absorption spectra of DOC were measured using a Specord 210 spectrophotometer (Analytik Jena). To approximate the quality of organic matter, the specific ultraviolet absorbance (SUVA_254_) and the spectral slope ratio (S_R_) were measured. The S_R_ was calculated as the ratio of spectral slopes from 275 to 295 nm, and from 350 to 400 nm (S_275–295_ to S_350–400_) [42]. The specific ultraviolet absorbance (SUVA_254_; m^2^ g^-1^) was measured by dividing the UV absorbance (m^-1^) measured at 254 nm by the DOC concentration (g m^-3^) [43]. The S_R_ and SUVA_254_ values indicate proportions of low molecular weight compounds and aromatic compounds, respectively, in the soil DOC [42, 43].

### Microbial and biochemical soil characteristics

Soil samples (10 g, adjusted to 60% of the water holding capacity, 12 replicates) were placed in glass bottles sealed with perforated parafilm and incubated at 10°C for 7 and 21 days. After the 7-day incubation, soils from 3 replicates were extracted (0.5 M K_2_SO_4_; extractant:soil ratio of 4:1; shaking for 60 min at horizontal shaker; centrifugation at 4000 g for 10 min; filtration through 0.45 μm glass fiber filters). Another 3 replicates were fumigated with chloroform for 24 h before extraction, performed as described for the non-fumigated samples. The non-fumigated extracts were analyzed for NH_4_ (NH_4ex_) and NO_3_ (NO_3ex_) using the FIA analyser, and together with the extracts from the fumigated samples also for total carbon and nitrogen concentrations, using an elemental analyser (LiqiTOC II, Elementar Analysensysteme GmbH, Germany). Microbial C (C_MB_) and N (N_MB_) were calculated as the differences between their respective concentrations in the fumigated and non-fumigated samples, and corrected using extraction efficiency factors of 0.38 for microbial C [44] and 0.54 for microbial N [45]. After the 21-day incubation, the remaining samples were either extracted (as described above) to determine the N mineralization potential (N mineralization and nitrification) [46] or fumigated and extracted to determine N_MB_. The net N mineralization and nitrification rates were calculated as the difference between the 21-day and 7-day concentrations of NH_4ex_ and NO_3ex_, respectively, divided by the number of days (14) [47]. All chemical and biochemical results are expressed on a dry weight (105°C) soil basis.

In addition to samples from the 6-week sampling obtained during this study, we also used data on DOC and C_MB_ concentrations and net nitrification rates measured at the same study plots using identical methods in late May 2004–2007 (Šantrůčková, unpublished data). These data represent the soil biogeochemistry prior to forest dieback.

### Calculations and statistical evaluations

A presence of increasing or decreasing trends in the 6-week time series was tested using a Mann-Kendall test (XLSTAT software). To test the homogeneity of the time series we used Petitt´s test (XLSTAT software). To test the presence of regular periodical trends in the 6-week time series, we calculated annual median values of respective parameters for each particular year of the study, and subtracted those medians from values measured in the respective year. Then we divided the values into categories according to season of sampling (winter, spring, summer, autumn), and evaluated the differences among the categories using Mann-Whitney test (STATISTICA 9 software). In the same way we compared also the seasons with presence or absence of the snow cover.

The differences in annual averages of chemical and biochemical parameters were compared using the non-parametric Mann-Whitney test (STATISTICA 9 software) due to the non-normal distribution of data. The differences in chemical and biochemical parameters between soil horizons at one plot (or between plots in similar horizons) were tested using non-parametric Wilcoxon test (STATISTICA 9 software). The correlations between measured parameters were calculated using MS Excel software.

### Permissions

Permission for access and the collection of all samples was issued by the Administration of the National Park and Protective Landscape Area of Šumava (permission numbers: SZ NPS 06537/2009/4-NPS 0921/2009 and SZ NPS 09741/2011/4-NPS 00099/2012).

## Results

### C and N concentrations

Despite the stable total C and N concentrations in the soil (Table 1), the concentrations of water-extractable DOC and all N forms displayed high temporal variation at both plots (S1 Table).

**Table 1:** Ranges and averages (in parentheses) of soil characteristics of the O and A horizons at the study plots in the Plešné (PL) and Čertovo (CT) catchments from 2008–2013.

	CT-O	CT-A	PL-O	PL-A
C (mol kg^-1^)	37–41 (39)	32–40 (37)	36–40 (39)	34–39 (37)
N (mol kg^-1^)	1.3–1.4 (1.3)	1.05–1.4 (1.2)	1.1–1.3 (1.2)	1–1.3 (1.2)
P (mmol kg^-1^)	25–30 (27)	20.4–27.5 (24)	23–32 (29)	19–28 (23)
C:N (molar ratio)	27–31 (29)	27–33 (31)	30–35 (31)	29–39 (35)
pH_KCl_	2.4–2.8	2.3–2.8	2.5–3.2	2.3–2.8

Abbreviations: total carbon (C), total nitrogen (N), total phosphorus (P).

**Fig 1:**
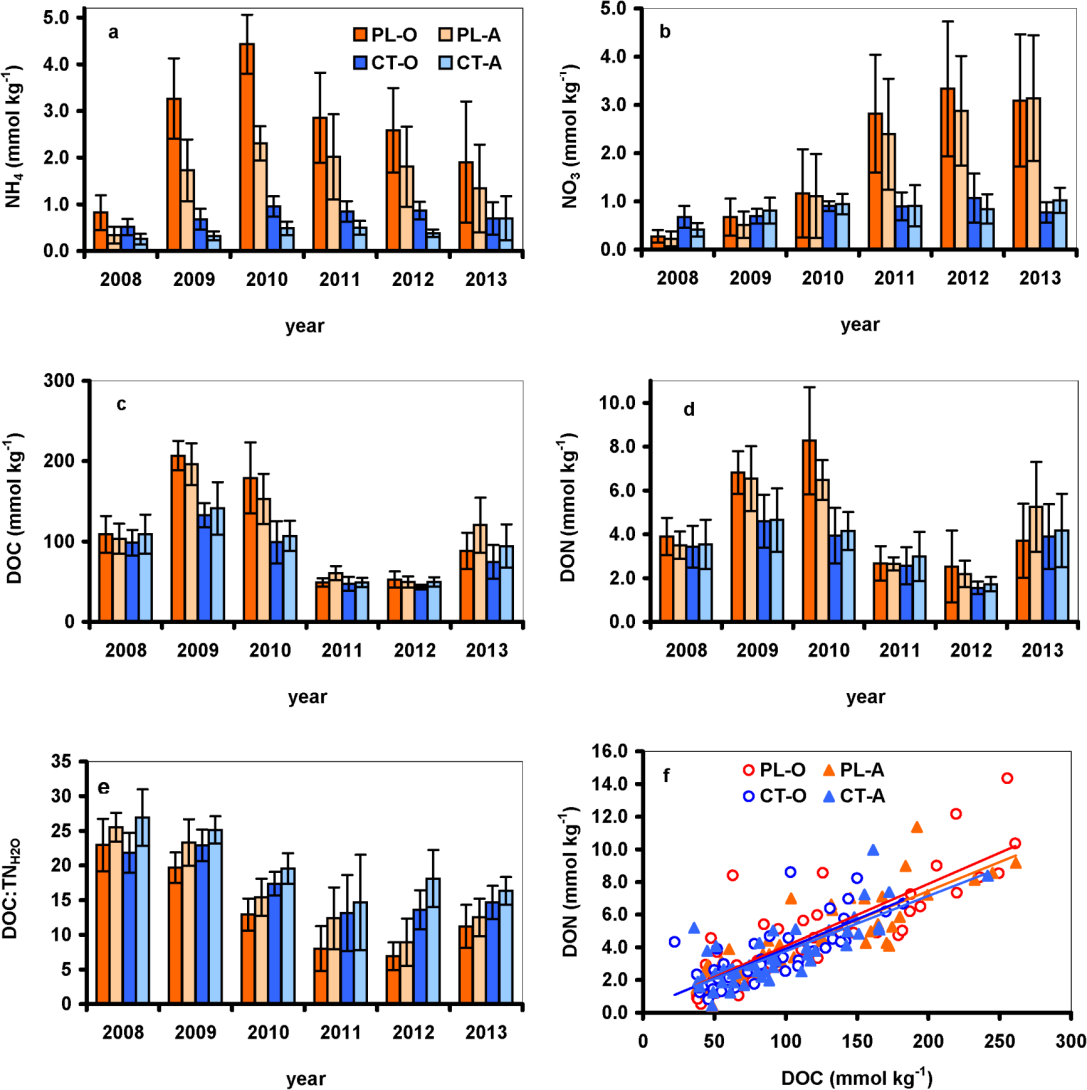
Annual average (n = 8–9 for each year) concentrations of (a) NH_4_, (b) NO_3_, (c) DOC, (d) dissolved organic N (DON), (e) annual average molar DOC:TN_H2O_ ratios, and (f) relationship between DOC and DON concentrations in water extracts from the O and A soil horizons at the Plešné (PL) and Čertovo (CT) plots. Data on concentrations from 2008–2010 are from Kaňa et al. (2013). Vertical bars denote 95% confidence intervals. Lines in Fig 1f represent linear regressions (PL-O: y = 0.04x + 0.27, *R* ^*2*^ = 0.70; PL-A: y = 0.04x + 0.35, *R* ^*2*^ = 0.75; CT-O: y = 0.04x + 0.29, *R* ^*2*^ = 0.58; CT-A: y = 0.03x + 0.38, *R* ^*2*^ = 0.68).

The annual average DOC concentrations were similar at both plots at the beginning of the study, varying from ~50 to ~100 mmol kg^-1^ in both horizons (Fig 1). Even though the DOC concentrations significantly increased (*p*<0.05) at both plots from 2008 to 2009, the DOC concentrations were significantly higher (*p*<0.01) at the PL plot than the CT plot. During the DOC peak in 2009, the respective annual average DOC concentrations were 235 and 214 mmol kg^-1^ in the O and A horizons at the PL plot, while only 140 and 148 mmol kg^-1^ at the CT plot (Fig 1, S1 Table). Then, the annual average DOC concentrations decreased to similar values at both plots in 2011 and increased slightly again in 2013 (Fig 1). The mean annual DON concentrations exhibited similar trends as DOC: (1) DON concentrations were similar at both plots in 2008 (~3.5 mmol kg^-1^), (2) peaked during the years 2009–2010, with higher values at the PL plot than the CT plot (~7–8 *vs*. 4–5 mmol kg^-1^), and then (3) decreased to ~1.5–2 mmol kg^-1^ at both plots, i.e., to values significantly lower than those in 2008 (Fig 1, S1 Table). The DON concentrations significantly (*p*<0.01) and linearly correlated with DOC at both plots (Fig 1), indicating that the DOC:DON ratios were similar there and did not change during the study.

The annual averages of NH_4_, and NO_3_ concentrations were similar at both study plots in 2008, but then showed different trends (Fig 1, S1 Fig). The concentration of dissolved inorganic N (DIN = NH_4_ + NO_3_) remained stable in the O horizon (~1.5 mmol kg^-1^) at the control CT plot throughout the study, and increased only slightly in the A horizon from 0.8 mmol kg^-1^ in 2008 to ~1.4 mmol kg^-1^ in 2009–2012. In contrast, the annual average DIN concentrations increased steeply at the disturbed PL plot, from 1.1 mmol kg^-1^ in 2008 to ~6 mmol kg^-1^ in 2011–2012 in the O horizon, and from 0.6 mmol kg^-1^ to 4.5 mmol kg^-1^ in the A horizon during the same period. The NH_4_ concentrations increased fourfold (from ~1 to ~4 mmol kg^-1^ in the O horizon, and from ~0.5 to ~2 mmol kg^-1^ in the A horizon) at the PL plot almost immediately after the forest dieback, with maximum values in 2010, and then continuously decreased to ~2 mmol kg^-1^ in the O horizon and to 1.3 mmol kg^-1^ in the A horizon in 2013 (Fig 1). The NH_4_ decrease was accompanied by an increase in NO_3_ concentrations after a period of relatively stable and low concentrations (2008–2010; Fig 1, S1 Fig, S1 Table).

As a result of changes in the concentrations of DOC and N forms, the molar DOC:TN_H2O_ ratio decreased from maximum values of 23–27 in 2010 to their minimum of 7–9 in 2012 in the PL soils (Fig 1E). A similar decline in the DOC:TN_H2O_ ratio was also observed in the CT soils, but was less pronounced: from 22 to 14 and from 27 to 18 in the O and A horizons, respectively (Fig 1E).

**Fig 2:**
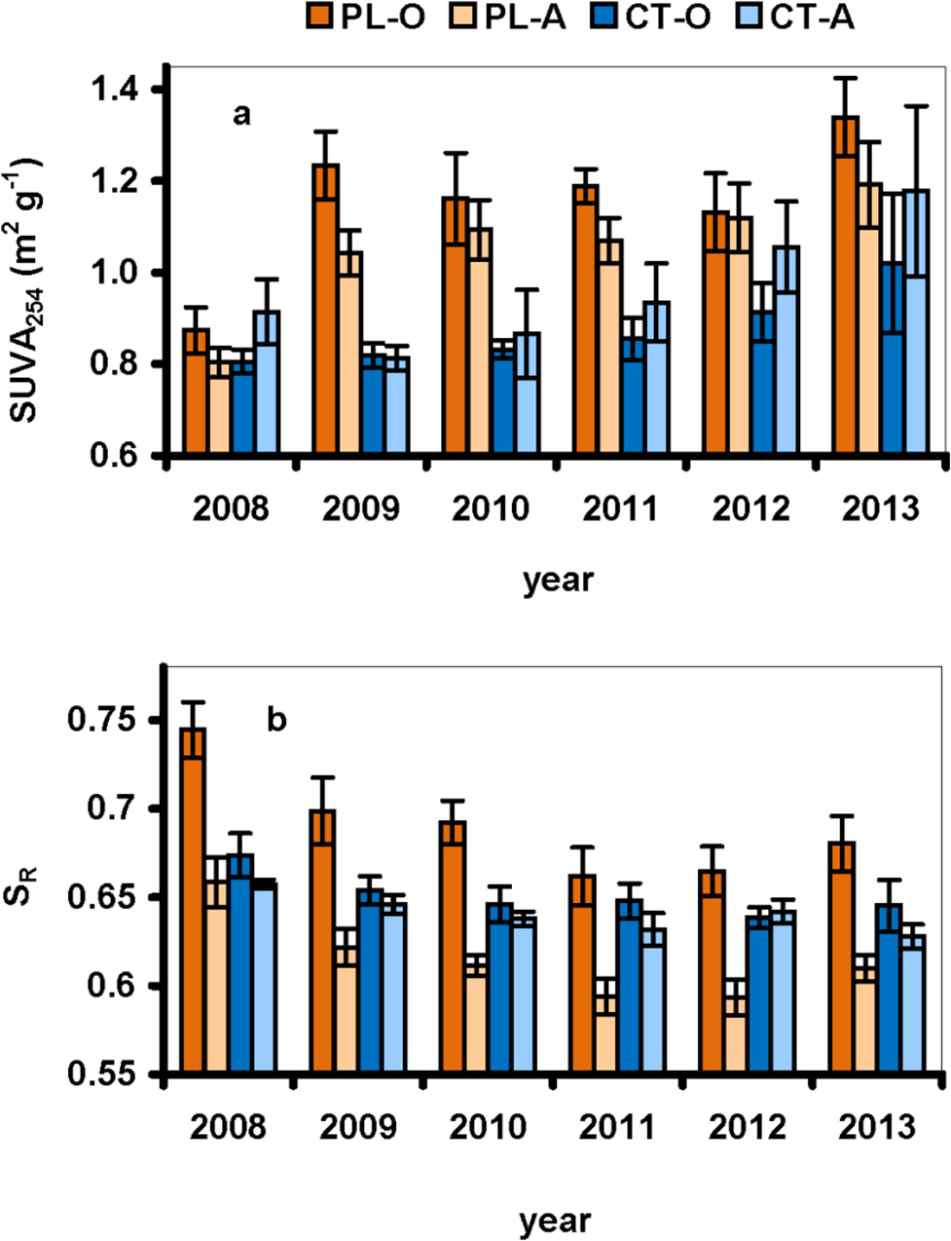
Annual average (n = 8–9 for each year) values of (a) the ratio between S_275–295_ and S_350–400_ slopes (S_R_), and (b) the specific absorbance of dissolved organic carbon at 254 nm (SUVA_254_) in water extracts from the O and A soil horizons at the Plešné (PL) and Čertovo (CT) plots.

Changes in the DOC concentrations were also accompanied by changes in DOC characteristics expressed by SUVA_254_ and S_R_ values. While the SUVA_254_ values were similar (0.8–0.9 m^2^ g^-1^) in both PL and CT soils in 2008, they increased significantly (to 1.2 and 1.1 m^2^ g^-1^ in the O and A horizons, respectively) after the forest dieback at the PL plot in 2009, and then remained stable. At the CT plot, the SUVA_254_ values displayed a consecutively increasing trend from 2011 in the O and A horizons (Fig 2A, S1 Fig). The S_R_ values were significantly (*p*<0.0001) higher at the PL than CT plot. The annual average S_R_ significantly (*p*<0.05) decreased at the PL plot until 2011 (from annual average values of 0.74 to 0.65 in the O horizon, and from 0.66 to 0.59 in the A horizon). At the CT plot, the S_R_ values were significantly decreasing in both horizons during the whole study (Fig 2B).

### Net N transformations and microbial biomass

**Fig 3:**
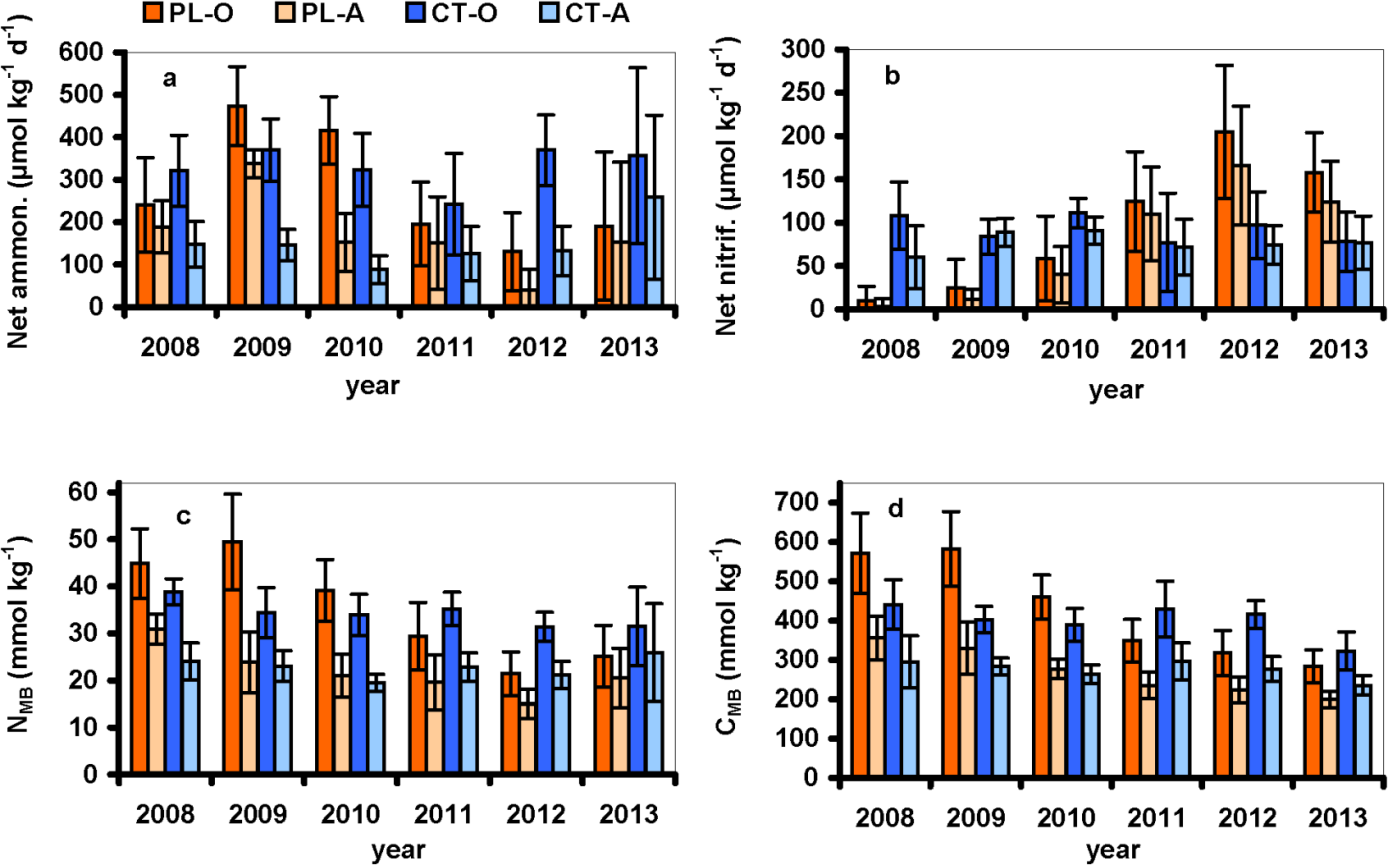
Annual average (n = 8–9 for each year) rates of (a) net ammonification and (b) net nitrification rates, and concentrations of (c) nitrogen (N_MB_) and (d) carbon (C_MB_) in soil microbial biomass in the O and A soil horizons at the Plešné (PL) and Čertovo (CT) plots. Vertical bars denote 95% confidence intervals.

**Fig 4:**
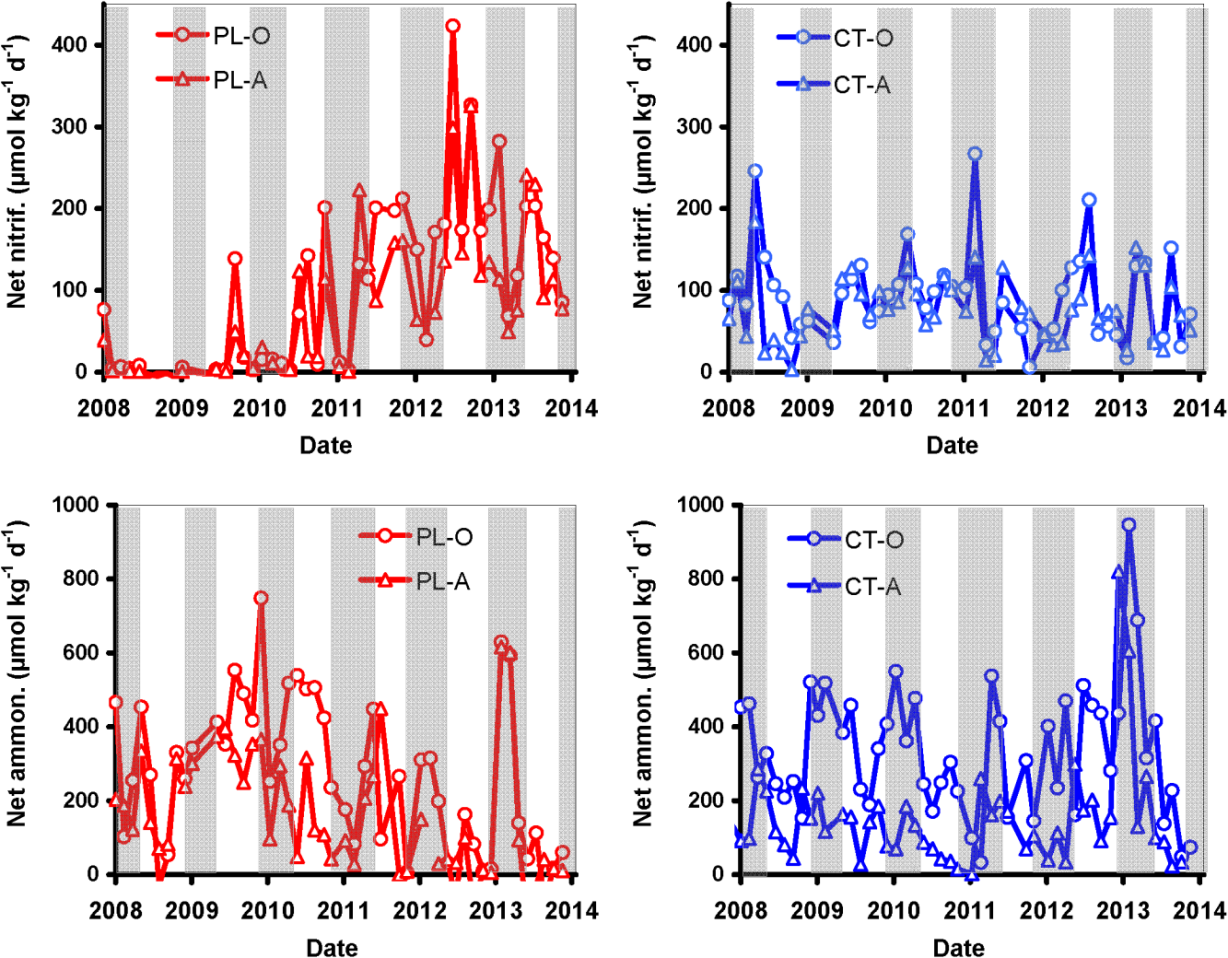
Temporal variability in net nitrification and net ammonification in the O and A soil horizons at the Plešné (PL) and Čertovo (CT) plots during the period 2008–2013. **Grey fields represent periods with snow cover.** Temporal variability of other soil parameters is shown in S1 and S2 and S3 Figs.

The annual average net ammonification rates were similar at both plots at the beginning of the study in 2008 (Fig 3A). Net ammonification was generally higher in the O horizon than in the A horizon (*p*<0.0001 for both plots), and displayed high temporal variability over the study period (Fig 4A), with values ranging from 30 to 540 μmol N kg^-1^ d^-1^ at the CT plot and from –70 to 750 μmol N kg^-1^ d^-1^ at the PL plot. Net ammonification varied between 0 and 300 and –90 and 450 μmol N kg^-1^ d^-1^ in the A horizon at the CT and PL plot, respectively (Fig 4, S1 Table). The trends in net ammonification rates differed between the study plots. The time series of net ammonification were homogeneous and without trend in both horizons at the CT plot (Fig 4 and S2 Table). In contrast, net ammonification rate was significantly (*p* = 0.0001) higher at PL plot from 2008 to 2010 than from 2011 to 2013. Annual averages of net ammonification continually decreased at the PL plot between 2009 and 2012 to values significantly (*p*<0.01) lower than in 2008 (Fig 3A). Net ammonification correlated significantly with the DOC and DON concentrations at the PL plot (*R* ^*2*^ = 0.70 and 0.66, respectively; *p*<0.01), but not at the CT plot.

**Fig 5:**
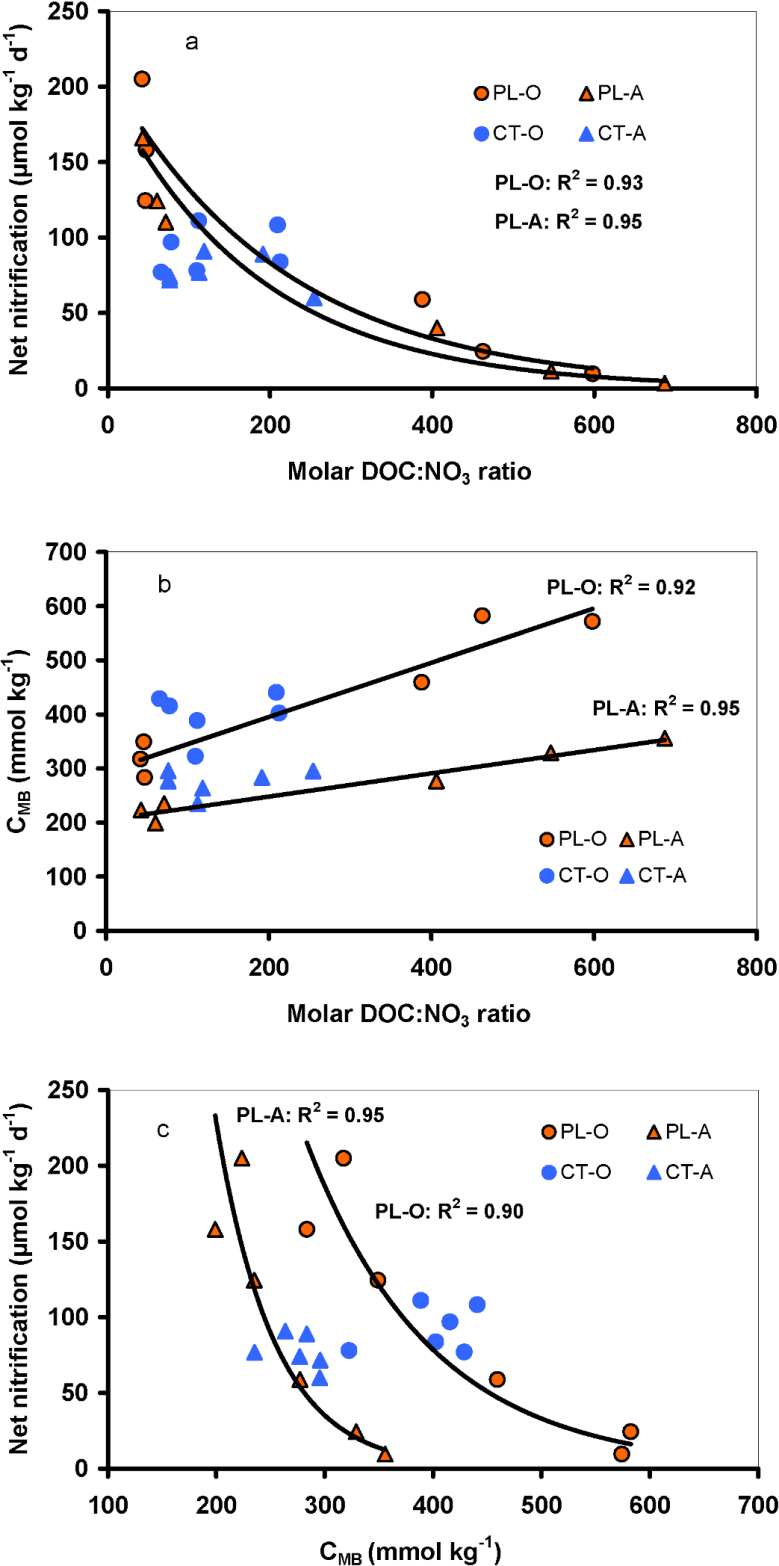
Relationships between annual average (n = 8–9 for each year) molar DOC:NO_3_ ratio in water extracts and (a) annual average net nitrification, (b) C in microbial biomass (C_MB_), and (c) between C_MB_ and net nitrification in the O and A soil horizons at the Plešné (PL) and Čertovo (CT) plots. Lines indicate statistically significant (*p*<0.01) correlations of respective parameters in soils from the PL plot.

Similarly to net ammonification, the temporal variability in net nitrification was high at both study plots (Fig 4). The net nitrification rates varied from 6 to 270 and –6 to 420 μmol N kg^-1^ d^-1^ in the O horizon at the CT and PL plots, respectively, and from 4 to 180 and –5 to 330 μmol N kg^-1^ d^-1^ in the A horizon at the CT and PL plots, respectively (Fig 4, S1 Table). The values of net nitrification did not follow any trend at the CT plot during the study period, while they significantly (*p*<0.0001) increased in both O and A horizons at the PL plot from 2010–2013 (Fig 3B, Fig 4), i.e., after the decrease in DOC concentrations (Fig 1C). This increase in net nitrification was accompanied by a parallel decrease in net ammonification, as well as N_MB_ and C_MB_ concentrations (Fig 3C and 3D). In particular, net nitrification increased when the DOC:NO_3_ molar ratio decreased below ~400, and then steeply increased as the ratios further decreased (Fig 5A).

The N_MB_ concentrations were higher by one order of magnitude than TN_H2O_ (S1 Table), ranging from 16–68 and 8–38 mmol kg^-1^ in the PL O and A horizons, respectively. The mean annual N_MB_ concentrations significantly (*p*<0.01) decreased in the PL soils between 2009 and 2012: from ~50 and ~30 to ~20 and ~15 mmol kg^-1^ in the O and A horizons, respectively (Fig 3C). The N_MB_ concentrations at the CT plot varied within ranges similar to the PL plot (S1 Table), but their annual averages were relatively stable (Fig 3C).

In concert with N_MB_, we observed a significant (*p*<0.0001)decrease in the C_MB_ concentrations at the PL plot, with annual averages decreasing from 574 to 314 and from 356 to 214 mmol kg^-1^ in the O and A horizons, respectively, from 2008–2013. In contrast, the C_MB_ concentrations remained stable at the CT plot throughout the study (Fig 3D). The decreases in C_MB_ and N_MB_ concentrations in the PL soil horizons were related (*p*<0.01) to the increase in net nitrification rates (shown only for C_MB_ in Fig 5C). The molar C_MB_:N_MB_ ratios were similar at both study plots, averaging ~12 and ~13 in the O and A horizons, respectively, and did not exhibit any trends (not shown).

### Seasonal trends

The investigated chemical and biochemical parameters did not show any general seasonal trends (Table 2). For example, the net nitrification rates were significantly (*p* = 0.049) higher in summer than in winter period in the PL O horizon. Similar trend was not, however, observed in the PL A horizon. Moreover, the net nitrification rates were significantly (*p* = 0.047) lower in winter than in autumn in the CT O horizon. The DOC concentrations were significantly higher in the CT soils during winter and spring than in summer. In contrast, there were no significant differences in DOC concentrations among seasons in PL soils. For more details on time series of measured chemical and biochemical parameters see SI (S1 and S2 and S3 Figs) and for their significances S2 Table.

**Table 2:** Differences in soil biochemical and chemical parameters among seasons (spring, Sp; summer, S; autumn, A; and winter, W) in the O and A horizons at the Plešné (PL) and Čertovo (CT) plots from 2008–2013. Different letters in superscript indicate statistically significant differences (p<0.05) among seasonal values (a–lower value, b–higher value, n.s.–not significant); significantly differing seasons are in bold.

	PL-O	PL-A	CT-O	CT-A
**Net nitrif.**	**W** ^**a**^ Sp^ab^ **S** ^**b**^ A^ab^	n.s.	**W** ^**b**^ Sp^ab^ S^ab^ **A** ^**a**^	n.s.
**Net ammon.**	n.s.	n.s.	**W** ^**b**^ Sp^ab^ **S** ^**a**^ A^ab^	n.s.
**NO**_**3**_	W^ab^ **Sp** ^**a**^ **S** ^**b**^ A^ab^	**W** ^**a**^ Sp^ab^ **S** ^**b**^ A^ab^	n.s.	n.s.
**NH**_**4**_	n.s.	**W** ^**a**^ Sp^ab^ **S** ^**b**^ A^ab^	n.s.	n.s.
**DIN**	W^ab^ **Sp** ^**a**^ **S** ^**b**^ A^ab^	W^ab^ **Sp** ^**a**^ **S** ^**b**^ A^ab^	n.s.	n.s.
**DOC**	n.s.	n.s.	**W** ^**b**^ **Sp** ^**b**^ **S** ^**a**^ A^ab^	**W** ^**b**^ **Sp** ^**b**^ **S** ^**a**^ A^ab^
**N**_**MB**_	n.s.	n.s.	n.s.	**W** ^**a**^ Sp^ab^ S^ab^ **A** ^**b**^

When we compared seasons with and without snow cover, we found higher net nitrification rates (*p*<0.05) and DIN concentrations (*p*<0.05) in the PL A horizon and higher NH_4_ concentrations (*p*<0.01) in the PL O horizon during seasons without snow, while significantly higher DOC concentrations in the CT soils during seasons with snow cover (*p*<0.01 and *p*<0.001 for the O and A horizon, respectively).

## Discussion

### C and N forms

**Fig 6:**
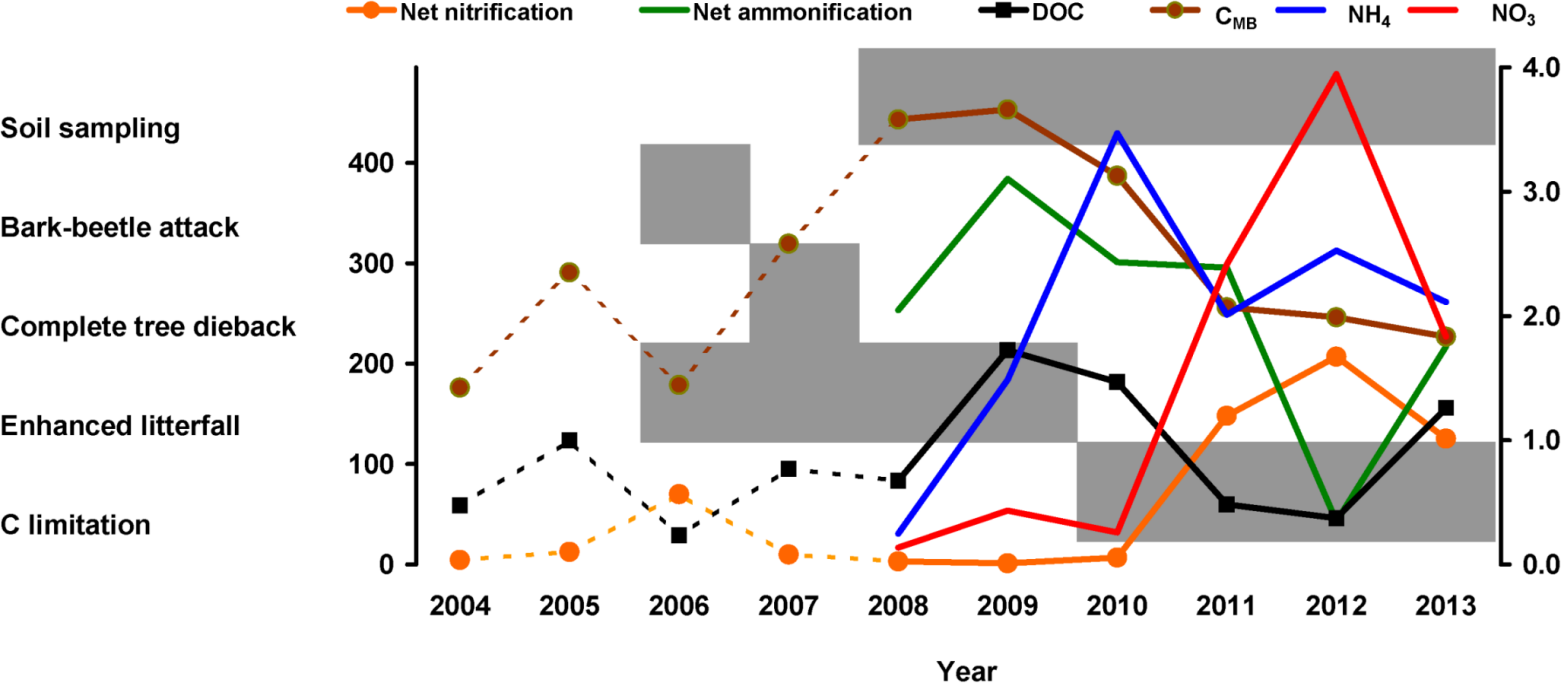
A simplified scheme of the main changes in C and N cycling along with the timeframes of major ecosystem changes (grey fields) at the Plešné plot, affected by the bark beetle infestation. The lines show mass weighted means of spring values of the respective variables in the O and A soil horizons. The left Y axis shows rates of net ammonification and net nitrification (μmol kg^-1^d^-1^), C in microbial biomass (C_MB_; mmol kg^-1^), and DOC concentrations in water extracts (mmol kg^-1^). The right Y axis shows concentrations of NH_4_ and NO_3_ (mmol kg^-1^) in water extracts. Values for the years 2004–2007 are based on regular annual spring samplings (late May) at the study plot (Šantrůčková, unpublished data). Similar scheme for unaffected CT plot is given in SI (S4 Fig).

The forest dieback at the PL plot brought about elevated C and nutrient inputs to soils due to enhanced litterfall [6,24]. Annual flux of C in the litterfall from trees attacked by bark beetle represented 31% of the C pool in the O horizon at the PL plot, while that at the control CT plot was only ~9% [24]. Nutrient input in the PL litterfall was enhanced in a range similar to C. These elevated C and nutrient inputs increased almost immediately soil concentrations of DOC, DON, microbial biomass, and inorganic N forms, as well as net ammonification at the PL plot (Fig 6) compared to the CT plot (S4 Fig), namely in the uppermost horizon, which was directly affected by litterfall. The increase in net nitrification and NO_3_ concentration was delayed 3 years, until the concentrations of DOC in soils abruptly decreased at the PL plot. This decrease was followed by slower decrease in microbial biomass, ammonification, and NH_4_ concentration (Fig 6). This decrease in DOC lasted for two years (2011 and 2012).

Concentrations of DOC and DON at the CT plot followed similar trends (but not so distinct) as at the PL plot. They peaked during the years 2009–2010 and then decreased during 2011–2012. Annual average DOC concentrations linearly correlated with precipitation in the O and A horizons at both plots, while correlation between precipitation and net ammonification was significant only in the O horizons (S5 Fig). The decrease in soil DOC concentrations in 2011–2012 thus primarily reflected low precipitation in these years. Thus, we suppose that the observed trends in DOC concentrations were primarily affected by climate at both plots, and accentuated by high litterfall at the PL plot.

It has been shown that soil DOC concentrations are closely related to organic matter mineralization [48]. In our study, soil DOC concentrations correlated with ammonification and microbial biomass at the infested PL plot but not at the CT plot. This difference was probably caused by different quality of litterfall after the infestation of the PL forest. The forest dieback resulted in the elevated input of fresh litter with lower C:N:P ratios than soil organic matter [24], which increased organic matter availability for microbial processes and accelerated mineralization. Under such conditions, microbes probably preferentially consumed easily available compounds and left behind more complex organic matter.

We observed increases in the proportion of aromatic compounds (increasing SUVA_254_; [43,49]) and high-molecular DOC compounds (a decreasing ratio of S_275–295_ to S_350–400_; [42]) in the total DOC pool during the study (Fig 2). The changes in quantity and quality of soil DOC could be also partially connected to soil pH increase at the PL plot in 2008–2010 [6]. It was shown during field experiments that DOC mobility in organic soils increased with pH increase [50], and the DOC composition shifted toward a larger sized matter, with higher proportion of aromatic and hydrophobic compounds [51].

In our study, post-disturbance increase in NO_3_ concentrations and net nitrification were delayed by 3 years after the elevated net ammonification at the PL plot (Fig 6). Their increases started when mineralization rate and DOC concentration decreased. Similarly, Huber et al. [15] have documented a rapid increase in DOC and NH_4_ concentrations in spruce forest soils in the nearby Bavarian Forest two years after a forest dieback, followed by their simultaneous decrease and an increase in NO_3_ concentrations in soil solutions. Kauffman et al. [52] also reported low concentrations of NO_3_ in drainage water from forest soils after clear-cutting until the DOC concentrations started to decrease. The decrease in DOC concentrations was then followed by increasing NO_3_ leaching. Moreover, it has been shown that ecosystems with higher pools of organic C lose less NO_3_ [53], which is consistent with the low flux of labelled N into the nitrate pool in Bohemian Forest soils with high C availability [54]. Such relationships support the theory that resource stoichiometry strongly couples C and N cycling in terrestrial ecosystems [30].

In contrast to the disturbed PL plot, the N uptake by unaffected forest remained probably stable at the control CT plot. This plot did not receive elevated litterfall [24] and, accordingly, microbial biomass, net ammonification and nitrification exhibited homogeneous time series there during the whole study (Fig 4, S2 Table). The NH_4_ concentrations remained low (S1 and S4 Figs), did not provide potential N source for nitrifiers, and, consequently, NO_3_ concentrations remained low at the CT plot, even during the period of decreased DOC concentrations in 2011–2012 (Fig 1).

The successive increase in net ammonification and then nitrification rate after forest dieback indicate an excess of inorganic N production over microbial N consumption (assimilation) and reduction (denitrification, dissimilatory nitrate reduction to ammonium–DNRA)[10,55]. The rapid increase in net ammonification immediately after the forest dieback, as well as the delayed increase in net nitrification, was apparently interconnected with the elevated availability of C for microbial processes during organic matter mineralization and, consequently, with the development of microbial biomass benefiting from this change. The net ammonification rates positively correlated with DOC and DON concentrations, and since 2009 also with N_MB_ concentrations and negatively with net nitrification rates (not shown). This indicates a fast microbial decomposition of N-rich organic compounds, N incorporation into N_MB_, and relatively small ammonium consumption in nitrification processes at the disturbed PL plot at the study beginning. On the contrary, the lack of correlation between net nitrification rates and ammonium concentrations shows that nitrification was not limited by ammonium deficiency in the soil. This implies that nitrification was limited by other factors in the early stage after forest dieback. The increase in nitrification (Fig 3B) immediately after the decrease in DOC concentrations (Fig 1C) and the reduced microbial biomass (Figs 3C, 3D, and 5C) suggest constraints of nitrification in a C-rich environment, with fast growing microbial communities after the forest dieback. A limitation of NO_3_ reduction processes by subsequent low DOC availability cannot be excluded, because these processes were not measured. We assume, however, that microbial NO_3_ consumption and reduction were too small under the low-DOC conditions to mitigate the NO_3_ excess in the soil. At the control CT plot, the values of microbial biomass and rates of net ammonification were independent of DOC and DON concentrations. This may imply microbial consumption of NH_4_ or the limitation of microbial decomposition activity by another factor (e.g., by low P availability [6]).

### C-limitation

Forest ecosystems are usually N-limited [56]. This is because microbes are richer in N than plant litter, which they use as the main C, energy, and nutrient resource, and they take up most of the organic nitrogen from decaying plant debris [57]. Trees support the development of mycorrhiza by available C in plant assimilates to obtain more N and other nutrients, and in this way accelerate the decay of soil organic matter and release of extra N. This general behaviour is altered in N-saturated and disturbed forests. In N-saturated ecosystems, trees are supplied with mineral N and decrease the allocation of plant assimilates to fine roots and mycorrhiza [58]. This results in a decrease in tree root biomass and, consequently, a decreasing biomass of ectomycorrhizal associations [59]. The declining pool of bioavailable DOC is accompanied by a progressively increasing NO_3_ availability in soils [30], indicating that the ecosystem shifts towards a break point when N mineralization exceeds the immobilization of mineral N forms by microbial and plant communities [60]. After a forest disturbance and/or dieback, the C supply from tree roots ceases and mycorrhizal associations diminish [61]. In an early phase after a forest dieback, however, the lack of C flux in plant assimilates may be offset by C flux from elevated litterfall [24], even though with different quality. As a result, mycorrhizal fungi can be replaced by saprotrophs, and plant litter is rapidly decomposed. After the most easily available organic compounds are mineralized, the litter decomposition and production of DOC available for soil microorganisms cease, and the microbial community becomes C-limited. Then, N mineralization can exceed N immobilization.

The elevated DOC availability temporarily mitigated N-saturation at the PL plot in 2009–2010, while the DOC decrease in the following 3–4 years brought about the symptoms of N-saturation at this plot. The similarity between the latter stage, following the forest dieback, and the effects of N-saturation due to formerly high N depositions is apparent when net nitrification rates at the PL (N-saturated, with forest dieback) and CT (N-saturated) plots are plotted against the DOC:NO_3_ ratios (Fig 5A). The data from both plots fit nonlinear negative correlations, but the PL data occur in the extreme positions in these relationships. Such a nonlinear negative relationship between the soil DOC:NO_3_ ratio and nitrification in a wide scale of soils was presented by Taylor and Townsend [30]. We observed a similar pattern, but with higher values of the DOC:NO_3_ ratio than in their study: the net nitrification increased when the DOC:NO_3_ ratio decreased to <400, and the increase was further accelerated when the DOC:NO_3_ ratio decreased to values around 50 at the PL plot (Fig 5A). The points with high DOC:NO_3_ ratios are from samples taken early after the forest dieback, when microbial biomass was high (Fig 5B). In contrast, the points with the lowest DOC:NO_3_ ratios are from the period after the decrease in DOC concentrations and microbial biomass, when nitrification rates increased (Fig 5A and 5B).

Our results support the hypothesis that the post-disturbance increase in C availability in N-saturated sites can cause shifts in the microbial community, leading to a transition from C-limitation to C excess, elevated microbial uptake of N, and the period of low NO_3_ concentrations in soils [29]. Then, after the restoration of the C-limited conditions, less N is assimilated by microbial biomass due to the C deficiency, surplus NH_4_ remains for proliferating nitrifying microbes, and NO_3_ production increases. The reduced C availability also limits energy demanding denitrification and DNRA. Consequently, the disequilibrium between NO_3_ production and consumption is shifted in favour of NO_3_ production, and more NO_3_ accumulates in the soil. In the first years after the forest dieback, the DOC concentrations and C availability were high at the PL plot, while net nitrification and NO_3_ concentrations were low, reflecting either the relatively higher importance of NO_3_ consumption (N assimilation, denitrification and DNRA) than production or low gross nitrification. We assume that the latter process is more plausible, and that slowly growing nitrifiers were outcompeted by rapidly growing heterotrophic microbes, similarly to the heterotrophic competition hypothesis [62]. Such presumed low gross nitrification in years with high DOC availability is also supported by Tahovská et al. [54], who measured negligible gross nitrification in the PL soils in spring 2008–2010, while gross ammonification was very high.

Our results thus indicate that the PL soil microbial community has become progressively more C-limited due to decreasing DOC concentrations since 2010, and less favorable conditions for heterotrophic decomposers has diminished NO_3_ consumption in the soil and accelerated the development of slowly growing autotrophic nitrifiers. Thus, the continuously decreasing C_MB_ and N_MB_ concentrations at the PL plot (Fig 3C and 3D) could be partly explained by the assumed decrease in heterotrophic microorganisms due to the reduced C availability. The close negative correlation between the microbial biomass and net nitrification (Fig 5C) then probably indicates the promotion of nitrifiers after the reduced biomass of heterotrophs. Presumable peak of heterotrophic activity after the forest dieback and the elevated C input to N-saturated plot was thus followed by a period of re-established C-limitation (under the conditions of still elevated NH_4_ concentrations, Fig 1A), increased activity of autotrophic nitrifying microbes, and elevated nitrate concentrations in soil.

## Conclusions

Our study has documented the importance of the organic C for nitrate leaching from soils after forest dieback. Net ammonification increased sharply together with DOC concentrations in soils almost immediately after the forest dieback, while net nitrification increased after a delay of ~3 years. The initial peak of mineralization together with high DOC concentrations resulted in elevated NH_4_ concentrations, but low net nitrification and NO_3_ concentrations. After the decreased DOC (but still elevated NH_4_) concentrations, NO_3_ production increased. Increase in net nitrification together with lower nitrate consumption due to decreased microbial biomass and absence of trees resulted in increased NO_3_ concentrations in soils. We propose that the main changes in soil biochemistry after the forest dieback were associated with a transition of the soil microbial community from C excess to C-limitation.

## Supporting Information

S1 Fig Temporal variability in concentrations of N forms. Temporal variability in concentrations of NO_3_, NH_4_, and dissolved organic N (DON) in the O and A soil horizons at the Plešné (PL) and Čertovo (CT) plots during the period 2008–2013. Grey fields represent seasons with snow cover.
(TIF)

S2 Fig Temporal variability in concentrations of C and N in microbial biomass. Temporal variability in concentrations of C and N in microbial biomass (C_MB_ and N_MB_) in the O and A soil horizons at the Plešné (PL) and Čertovo (CT) plots during the period 2008–2013. Grey fields represent seasons with snow cover.
(TIF)

S3 Fig Temporal variability in concentrations of DOC, SUVA_254_ and S_R._
Temporal variability in concentrations of dissolved organic carbon (DOC), and of SUVA_254_ and S_R_ values in the O and A soil horizons at the Plešné (PL) and Čertovo (CT) plots during the period 2008–2013. Grey fields represent seasons with snow cover.
(TIF)

S4 Fig Scheme of annual changes in C and N cycling at the Čertovo plot. A simplified scheme of annual changes in C and N cycling at the Čertovo plot, unaffected by the bark beetle infestation. The lines show mass weighted means of spring values of the respective variables in the O and A soil horizons (mass weighted means). The left Y axis shows rates of net ammonification and net nitrification (μmol kg^-1^d^-1^), C in microbial biomass (C_MB_; mmol kg^-1^), and DOC concentrations in water extracts (mmol kg^-1^). The right Y axis shows concentrations of NH_4_ and NO_3_ (mmol kg^-1^) in water extracts. Values for the years 2004–2007 are based on regular annual spring samplings (late May) at the study plot (Šantrůčková, unpublished data).
(TIF)

S5 Fig Relationships between precipitation, DOC, and net ammonification. Relationships between the sum of precipitation during the seasons without snow cover (Kopáček, unpublished data) and annual average DOC concentrations and net ammonification rates in the O and A soil horizons at the Plešné (PL) and Čertovo (CT) plots during the period 2008–2013. Red and blue lines represent linear regression between the sum of precipitation and annual average DOC concentrations at the PL and CT plot, respectively. Black line represents linear correlation between the sum of precipitation and annual average net ammonification rates in the O horizons at both plots.
(TIF)

S1 Table Ranges and annual averages of measured soil parameters. Annual ranges and averages (in brackets) of concentrations of dissolved organic carbon (DOC), N forms, and of C and N concentrations in soil microbial biomass (C_MB_, N_MB_), total N in H_2_O extract (TN_H2O_), dissolved organic nitrogen (DON), ammonium (NH_4_), nitrate (NO_3_) and net ammonification and nitrification rates in the O and A soil horizons at Čertovo (CT) and Plešné (PL) plots. Different letters in superscript indicate significant differences (*p*<0.05) among annual averages. The data on DOC, TN_H2O_, DON, NO_3_, and NH_4_ in period 2008–2010 are from Kaňa et al. [6].
(DOC)

S2 Table Significance of trends in data time series. Soil parameters with statistically significantly increasing or decreasing trends from 2008–2013 (see S1 Fig) in the O and A horizons at the Plešné (PL) and Čertovo (CT) plots. τ –Kendall´s tau, ↓ - decrease of respective parameter from 2008 to 2013, ↑ - increase of respective parameter from 2008 to 2013, n.s.–not significant (*p*>0.05).
(DOC)
