# Supplementary figures and images for: Excess of Organic Carbon in Mountain Spruce Forest Soils after Bark Beetle Outbreak Altered Microbial N Transformations and Mitigated N-Saturation
Source: PLoS One. 2015 Jul 31;10(7):e0134165. doi: 10.1371/journal.pone.0134165 (PMC4521819; doi:10.1371/journal.pone.0134165)

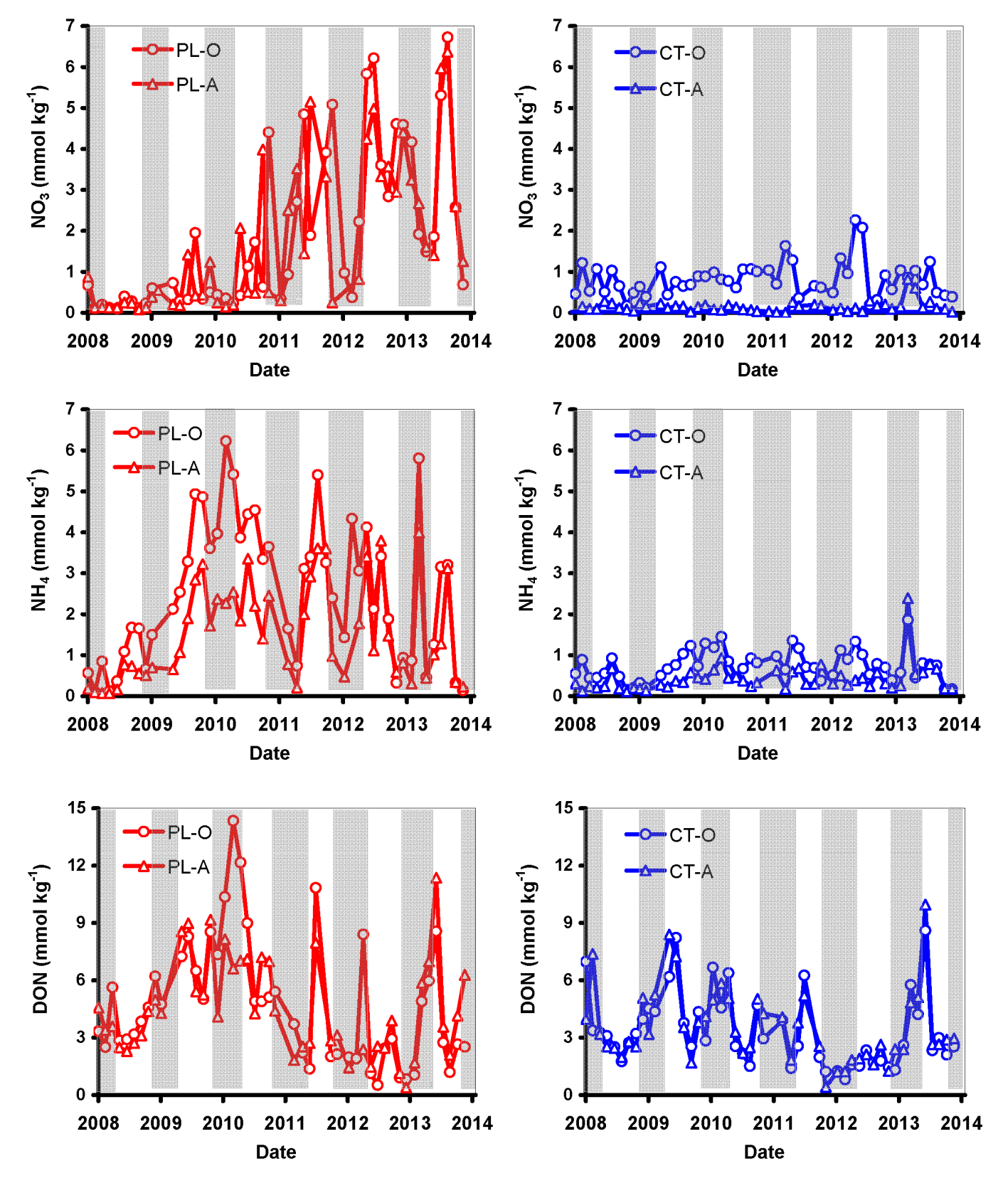

Supplement: S1 Fig — Temporal variability in concentrations of NO3, NH4, and dissolved organic N (DON) in the O and A soil horizons at the Plešné (PL) and Čertovo (CT) plots during the period 2008–2013. Grey fields represent seasons with snow cover. (TIF) [file pone.0134165.s001.tif]

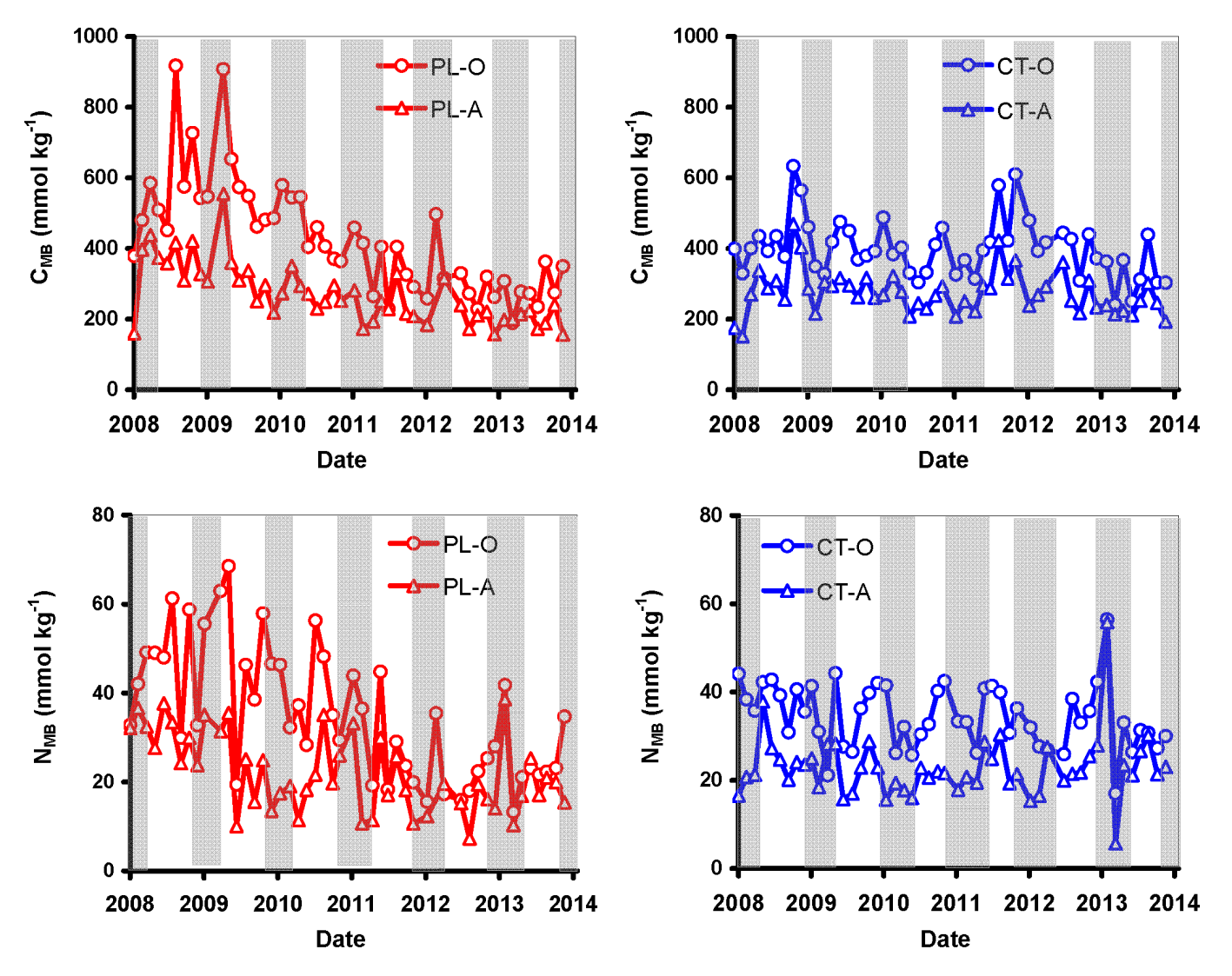

Supplement: S2 Fig — Temporal variability in concentrations of C and N in microbial biomass (CMB and NMB) in the O and A soil horizons at the Plešné (PL) and Čertovo (CT) plots during the period 2008–2013. Grey fields represent seasons with snow cover. (TIF) [file pone.0134165.s002.tif]

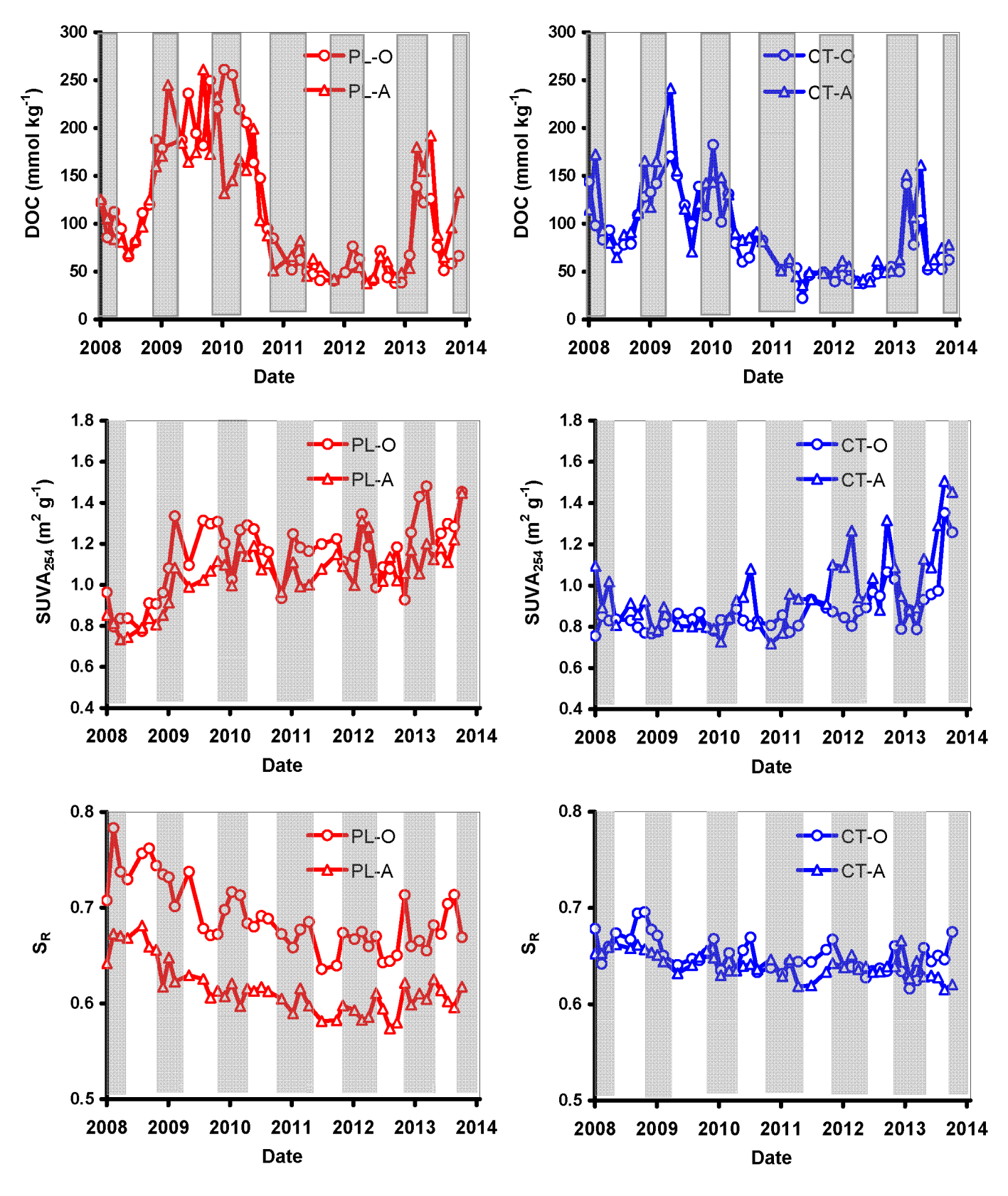

Supplement: S3 Fig — Temporal variability in concentrations of dissolved organic carbon (DOC), and of SUVA254 and SR values in the O and A soil horizons at the Plešné (PL) and Čertovo (CT) plots during the period 2008–2013. Grey fields represent seasons with snow cover. (TIF) [file pone.0134165.s003.tif]

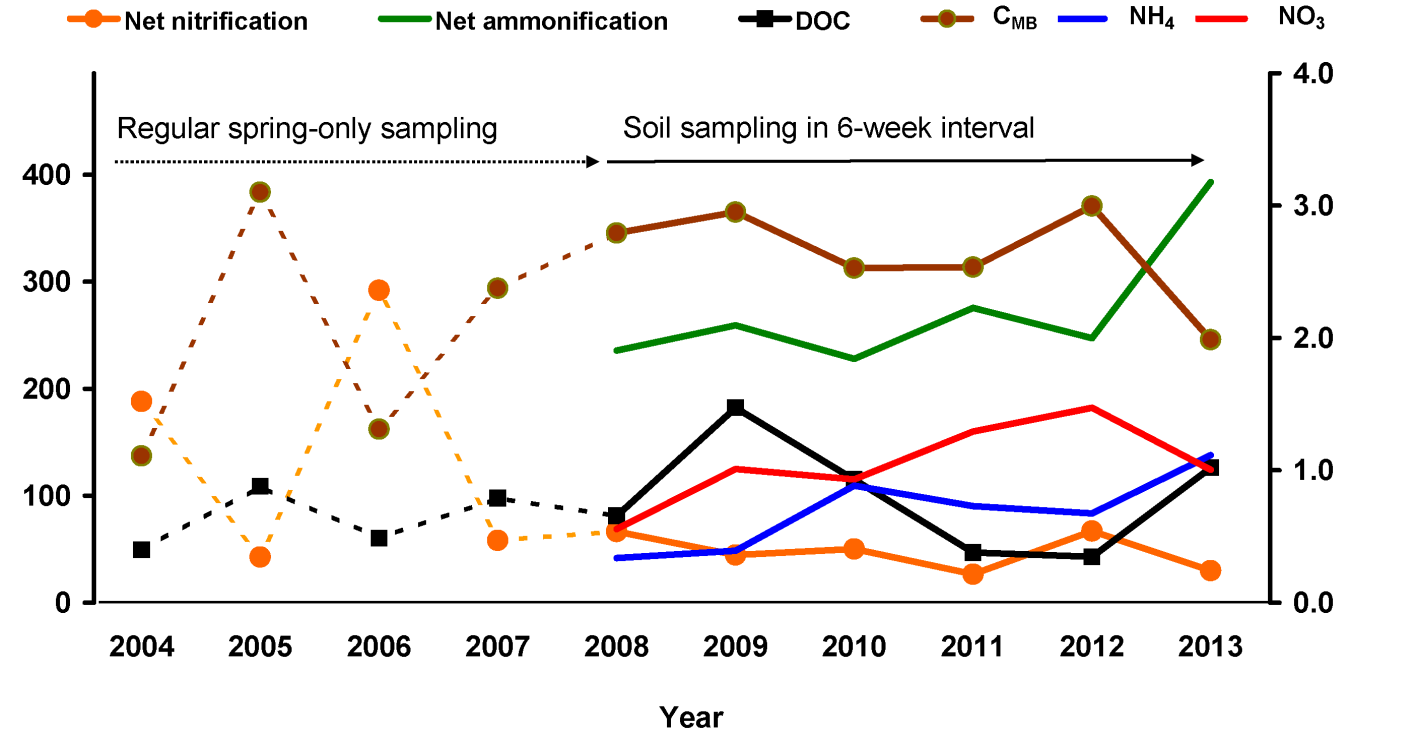

Supplement: S4 Fig — A simplified scheme of annual changes in C and N cycling at the Čertovo plot, unaffected by the bark beetle infestation. The lines show mass weighted means of spring values of the respective variables in the O and A soil horizons (mass weighted means). The left Y axis shows rates of net ammonification and net nitrification (μmol kg-1d-1), C in microbial biomass (CMB; mmol kg-1), and DOC concentrations in water extracts (mmol kg-1). The right Y axis shows concentrations of NH4 and NO3 (mmol kg-1) in water extracts. Values for the years 2004–2007 are based on regular annual spring samplings (late May) at the study plot (Šantrůčková, unpublished data). (TIF) [file pone.0134165.s004.tif]

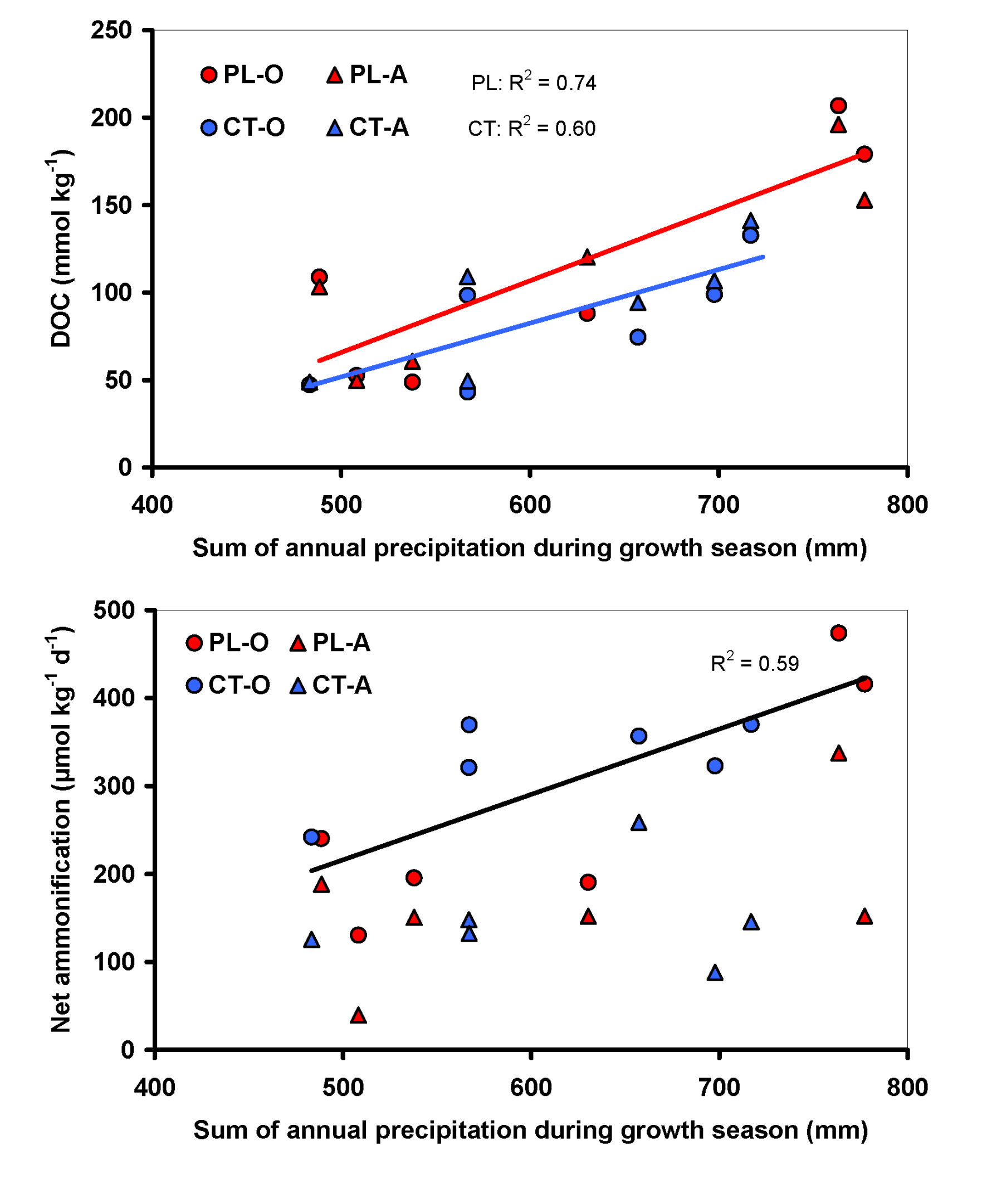

Supplement: S5 Fig — Relationships between the sum of precipitation during the seasons without snow cover (Kopáček, unpublished data) and annual average DOC concentrations and net ammonification rates in the O and A soil horizons at the Plešné (PL) and Čertovo (CT) plots during the period 2008–2013. Red and blue lines represent linear regression between the sum of precipitation and annual average DOC concentrations at the PL and CT plot, respectively. Black line represents linear correlation between the sum of precipitation and annual average net ammonification rates in the O horizons at both plots. (TIF) [file pone.0134165.s005.tif]
