# Supplementary material for: Excess of Organic Carbon in Mountain Spruce Forest Soils after Bark Beetle Outbreak Altered Microbial N Transformations and Mitigated N-Saturation
Source: PLoS One. 2015 Jul 31;10(7):e0134165. doi: 10.1371/journal.pone.0134165 (PMC4521819; doi:10.1371/journal.pone.0134165)
Supplement: S1 Table — Annual ranges and averages (in brackets) of concentrations of dissolved organic carbon (DOC), N forms, and of C and N concentrations in soil microbial biomass (CMB, NMB), total N in H2O extract (TNH2O), dissolved organic nitrogen (DON), ammonium (NH4), nitrate (NO3) and net ammonification and nitrification rates in the O and A soil horizons at Čertovo (CT) and Plešné (PL) plots. Different letters in superscript indicate significant differences (p<0.05) among annual averages. The data on DOC, TNH2O, DON, NO3, and NH4 in period 2008–2010 are from Kaňa et al. [6]. (DOC) [file pone.0134165.s006.doc]

|  | **year** | **CT-O** | **CT-A** | **PL-O** | **PL-A** |
| --- | --- | --- | --- | --- | --- |
| **DOC** | 2008 | 74–144 (98)^b^ | 65–172 (109)^b^ | 66–187 (109)^b^ | 69–160(103)^b^ |
| mmol kg^-1^ | 2009 | 100–199 (140)^c^ | 71–242 (148)^c^ | 179–431 (235)^c^ | 165–366 (214)^c^ |
|  | 2010 | 60–182 (99)^b^ | 82–147 (108)^b^ | 95–261 (173)^c^ | 88–232 (153)^c^ |
|  | 2011 | 22–60 (47)^a^ | 36–63 (49)^a^ | 41–62 (49)^a^ | 45–82 (61)^a^ |
|  | 2012 | 38–55 (44)^a^ | 38–61 (50)^a^ | 38–76 (51)^a^ | 38–66 (50)^a^ |
|  | 2013 | 52–141 (75)^ab^ | 56–161 (94)^ab^ | 51–138 (88)^ab^ | 54–192 (120) ^b^ |
| **TN_H2O_** | 2008 | 3.5–8 (4.6)^ab^ | 2.7–8.2 (4.2)^b^ | 3.1–7.1 (4.9)^a^ | 2.6–5.6 (4.1)^a^ |
| mmol kg^-1^ | 2009 | 4.2–9.9 (6.4)^b^ | 2.7–10.4 (6)^b^ | 6.8–23.7 (10.8)^b^ | 5.4–17 (9.8)^c^ |
|  | 2010 | 3.2–8.9 (5.8)^b^ | 3.9–7.9 (5.6)^b^ | 9.1–20.9 (13.9)^c^ | 8.9–11.8 (9.9)^c^ |
|  | 2011 | 2.2–8.7 (4.7)^ab^ | 1.8–7.4 (4.5)^ab^ | 3.9–19.1 (9.1)^b^ | 3.1–14.4 (7.8)^bc^ |
|  | 2012 | 2.3–5.4 (3.4)^a^ | 2–4 (3)^a^ | 4.4–13.7 (8.2)^b^ | 2.2–9.6 (6.7)^b^ |
|  | 2013 | 2.7–10.1 (5.4)^ab^ | 3.7–11.1 (5.9)^b^ | 3.4–12.6 (8.7)^b^ | 5.2–13.8 (9.7)^bc^ |
| **DON** | 2008 | 1.8–7 (3.4)^b^ | 2–7.4 (3.5)^b^ | 2.5–6.2 (3.9)^b^ | 2.3–5 (3.5)^b^ |
| mmol kg^-1^ | 2009 | 2.5–8.2 (5)^b^ | 1.7–8.4 (4.9)^b^ | 4.7–8.5 (6.8)^c^ | 4.1–9.2 (6.5)^c^ |
|  | 2010 | 1.5–6.7 (3.9)^b^ | 2.2–5.8 (4.1)^b^ | 4.9–14.3 (8.3)^c^ | 4.3–8.1 (6.5)^c^ |
|  | 2011 | 1.2–6.3 (2.9)^ab^ | 0.4–5.2 (3)^ab^ | 1.4–10.8 (3.7)^ab^ | 1.8–8 (3.5)^ab^ |
|  | 2012 | 0.8–2.3 (1.5)^a^ | 1.2–2.6 (1.8)^a^ | 0.5–8.4 (2.3)^a^ | 0.4–3.9 (2)^a^ |
|  | 2013 | 2.1–8.6 (3.9)^b^ | 2.4–10 (4.2)^b^ | 1.1–8.6 (3.7)^ab^ | 1.7–11.4 (5.3)^bc^ |
| **NO_3_** | 2008 | 0.17–1.21 (0.68)^a^ | 0.04–0.71 (0.41)^a^ | 0.1–0.67 (0.25)^a^ | 0.08–0.88 (0.23)^a^ |
| mmol kg^-1^ | 2009 | 0.39–1.12 (0.73)^a^ | 0.42–1.76 (0.81)^a^ | 0.29–1.95 (0.74)^a^ | 0.18–1.42 (0.52)^a^ |
|  | 2010 | 0.61–1.07 (0.9)^a^ | 0.6–1.45 (0.94)^a^ | 0.24–4.41 (1.17)^ab^ | 0.15–4 (1.1)^ab^ |
|  | 2011 | 0.36–1.63 (0.9)^a^ | 0.43–2.12 (0.91)^a^ | 0.34–5.1 (2.8) ^b^ | 0.3–5.15 (2.4)^b^ |
|  | 2012 | 0.19–2.26 (1.01)^a^ | 0.34–1.67 (0.79)^a^ | 0.38–6.2 (3.5)^b^ | 0.26–5 (3.1)^b^ |
|  | 2013 | 0.39–1.24 (0.77)^a^ | 0.53–1.54 (1.02)^a^ | 0.69–6.7 (3.1)^b^ | 1.26–6.4 (3.1)^b^ |
| **NH_4_** | 2008 | 0.15–0.92 (0.52)^a^ | 0.12–0.68 (0.26)^a^ | 0.11–1.67 (0.8)^a^ | 0.07–0.77 (0.35)^a^ |
| mmol kg^-1^ | 2009 | 0.21–1.23 (0.72)^ab^ | 0.14–0.58 (0.32)^ab^ | 7.5–7.5 (3.8)^bc^ | 0.66–4.4 (2.06)^b^ |
|  | 2010 | 0.47–1.45 (0.96)^b^ | 0.25–0.93 (0.48)^b^ | 3.4-6.2 (4.4) ^c^ | 1.41–3.35 (2.31)^b^ |
|  | 2011 | 0.38–1.36 (0.85)^ab^ | 0.18–0.77 (0.5)^b^ | 0.74–5.4 (2.85)^b^ | 0.21–3.61 (2.02)^b^ |
|  | 2012 | 0.39–1.34 (0.81)^ab^ | 0.2–0.58 (0.36)^ab^ | 0.32–4.33 (2.4)^b^ | 0.48–3.8 (1.68)^b^ |
|  | 2013 | 0.17–1.86 (0.69)^ab^ | 0.18–2.4 (0.7)^ab^ | 0.14–5.8 (1.9)^ab^ | 0.23–4 (1.34)^b^ |
| **C_MB_** | 2008 | 330–633 (441)^b^ | 151–469 (295)^b^ | 379–918 (574)^c^ | 160–438 (356)^b^ |
| mmol kg^-1^ | 2009 | 328–476 (402)^ab^ | 216–417 (284)^b^ | 462–653 (536)^bc^ | 218–554 (329)^ab^ |
|  | 2010 | 304–487 (389)^ab^ | 208–322 (264)^ab^ | 264–580 (459)^b^ | 230–351 (277)^ab^ |
|  | 2011 | 315–610 (429)^b^ | 208–423 (296)^b^ | 229–459 (349)^a^ | 172–330 (235)^a^ |
|  | 2012 | 215–479 (396)^b^ | 218–361 (272)^ab^ | 229–497 (310)^a^ | 157–316 (214)^a^ |
|  | 2013 | 215–411 (314)^a^ | 193–301 (244)^a^ | 160–363 (274)^a^ | 140–240 (196)^a^ |
| **N_MB_** | 2008 | 31–44 (39)^b^ | 17–38 (24)^a^ | 30–61 (45)^cd^ | 24–38 (31)^c^ |
| mmol kg^-1^ | 2009 | 21–44 (34)^ab^ | 16–29 (23)^a^ | 23–68 (50)^d^ | 10–36 (24)^bc^ |
|  | 2010 | 26–42 (34)^ab^ | 16–23 (19)^a^ | 28–56 (39)^c^ | 11–35 (21)^b^ |
|  | 2011 | 26–41 (34)^ab^ | 18–30 (23)^a^ | 18–45 (29)^b^ | 8–33 (19)^ab^ |
|  | 2012 | 16–42 (33)^ab^ | 15–28 (20)^a^ | 16–35 (24)^a^ | 7–20 (14)^a^ |
|  | 2013 | 26–33 (30)^a^ | 21–30 (24)^a^ | 21-35 (24)^a^ | 15–25 (19)^ab^ |
| **Net** | 2008 | 153–521 (321)^a^ | 40–270 (140)^a^ | -29–466 (240)^a^ | 40–330 (170)^b^ |
| **ammonification** | 2009 | 189–518 (374)^a^ | 40–290 (170)^a^ | 350–750 (490)^b^ | 250–400 (320)^c^ |
| µmol kg^-1^ d^-1^ | 2010 | 172–550 (323)^a^ | 40–190 (90)^a^ | 190–540 (400)^b^ | 40–320 (160)^ab^ |
|  | 2011 | 33–537 (242)^a^ | 0–260 (140)^a^ | 100–420 (240)^ab^ | 10–450 (160)^ab^ |
|  | 2012 | 162–512 (370)^a^ | 40–300 (140)^a^ | -70–320 (130)^a^ | -90–150 (40)^a^ |
|  | 2013 | 49–946 (357)^a^ | 23–820 (259)^a^ | -72–629 (191)^ab^ | -137–616 (152)^ab^ |
| **Net** | 2008 | 42–246 (108)^a^ | 3.6–184 (60)^a^ | -6.2–77 (10)^a^ | -4.9–39 (3.2)^a^ |
| **nitrification** | 2009 | 36–150 (91)^a^ | 51–127 (91)^a^ | -1–139 (37)^a^ | -2.3–49 (10)^a^ |
| µmol kg^-1^ d^-1^ | 2010 | 78–169 (111)^a^ | 28–128 (91)^a^ | 3–201 (59)^ab^ | -1.8–124 (40)^ab^ |
|  | 2011 | 6–267 (77)^a^ | 15–141 (72)^a^ | 3–212 (124) ^b^ | 1.3–223 (110)^b^ |
|  | 2012 | 17–211 (98)^a^ | 27–143 (74)^a^ | 40–423 (205)^b^ | 64–326 (166)^b^ |
|  | 2013 | 29-152 (78)^a^ | 27–153 (77)^a^ | 69–282 (158)^b^ | 50–241 (124)^b^ |
