# Supplementary material for: Excess of Organic Carbon in Mountain Spruce Forest Soils after Bark Beetle Outbreak Altered Microbial N Transformations and Mitigated N-Saturation
Source: PLoS One. 2015 Jul 31;10(7):e0134165. doi: 10.1371/journal.pone.0134165 (PMC4521819; doi:10.1371/journal.pone.0134165)
Supplement: S2 Table — Soil parameters with statistically significantly increasing or decreasing trends from 2008–2013 (see S1 Fig) in the O and A horizons at the Plešné (PL) and Čertovo (CT) plots. τ –Kendall´s tau, ↓ - decrease of respective parameter from 2008 to 2013, ↑ - increase of respective parameter from 2008 to 2013, n.s.–not significant (p>0.05). (DOC) [file pone.0134165.s007.doc]

|  | trend | p value | τ |  |  | trend | p value | τ |
| --- | --- | --- | --- | --- | --- | --- | --- | --- |
| **net nitrification** |  |  |  |  | **net ammon.** |  |  |  |
| PL-O | ↑ | < 0.0001 | 0.519 |  | PL-O | ↓ | 0.006 | -0.275 |
| PL-A | ↑ | < 0.0001 | 0.526 |  | PL-A | ↓ | 0.0003 | -0.356 |
| CT-O | n.s. | 0.090 | -0.168 |  | CT-O | n.s. | 0.425 | -0.080 |
| CT-A | n.s. | 0.979 | 0.003 |  | CT-A | n.s. | 0.650 | -0.046 |
|  |  |  |  |  |  |  |  |  |
| **NO_3_** |  |  |  |  | **NH_4_** |  |  |  |
| PL-O | ↑ | < 0.0001 | 0.560 |  | PL-O | n.s. | 0.771 | 0.030 |
| PL-A | ↑ | < 0.0001 | 0.543 |  | PL-A | n.s. | 0.061 | 0.190 |
| CT-O | n.s. | 0.589 | 0.054 |  | CT-O | n.s. | 0.487 | 0.070 |
| CT-A | n.s. | 0.072 | 0.179 |  | CT-A | ↑ | 0.022 | 0.226 |
|  |  |  |  |  |  |  |  |  |
| **DOC** |  |  |  |  | **DON** |  |  |  |
| PL-O | ↓ | 0.001 | -0.328 |  | PL-O | ↓ | 0.036 | -0.212 |
| PL-A | ↓ | 0.025 | -0.229 |  | PL-A | n.s. | 0.220 | -0.127 |
| CT-O | ↓ | < 0.0001 | -0.385 |  | CT-O | ↓ | 0.024 | -0.225 |
| CT-A | ↓ | 0.001 | -0.337 |  | CT-A | n.s. | 0.164 | -0.140 |
|  |  |  |  |  |  |  |  |  |
| **C_MB_** |  |  |  |  | **N_MB_** |  |  |  |
| PL-O | ↓ | < 0.0001 | -0.592 |  | PL-O | ↓ | < 0.0001 | -0.454 |
| PL-A | ↓ | < 0.0001 | -0.560 |  | PL-A | ↓ | 0.001 | -0.328 |
| CT-O | n.s. | 0.061 | -0.184 |  | CT-O | ↓ | 0.025 | -0.220 |
| CT-A | n.s. | 0.088 | -0.167 |  | CT-A | n.s. | 0.616 | 0.050 |
|  |  |  |  |  |  |  |  |  |
| **SUVA_254_** |  |  |  |  | **S_R_** |  |  |  |
| PL-O | ↑ | 0.002 | 0.326 |  | PL-O | ↓ | < 0.0001 | -0.465 |
| PL-A | ↑ | < 0.0001 | 0.514 |  | PL-A | ↓ | < 0.0001 | -0.471 |
| CT-O | ↑ | < 0.0001 | 0.446 |  | CT-O | ↓ | 0.002 | -0.325 |
| CT-A | ↑ | 0.000 | 0.374 |  | CT-A | ↓ | < 0.0001 | -0.476 |
